# *Angelica keiskei* water extract Mitigates Age-Associated Physiological Decline in Mice

**DOI:** 10.1080/13510002.2024.2305036

**Published:** 2024-02-23

**Authors:** Huan Liu, Gang Wei, Tongxing Wang, Yunlong Hou, Bin Hou, Xiaoyan Li, Chao Wang, Mingzhe Sun, Min Su, Zhifang Guo, Lu Wang, Ning Kang, Mengnan Li, Zhenhua Jia

**Affiliations:** a Key Laboratory of State Administration of TCM (Cardio-Cerebral Vessel Collateral Disease), Shijiazhuang, People’s Republic of China; b Hebei Provincial Key Laboratory of Luobing, Shijiazhuang, People’s Republic of China; c National Key Laboratory for Innovation and Transformation of Luobing Theory, Shijiazhuang, People’s Republic of China; d College of Food Science & Nutritional Engineering, China Agricultural University, Beijing, People’s Republic of China; e Hebei Academy of Integrated Traditional Chinese and Western Medicine, Shijiazhuang, People’s Republic of China.; f High-Level TCM Key Disciplines of National Administration of Traditional Chinese, Shijiazhuang, People's Republic of China

**Keywords:** Oxidative stress, nature products, healthy aging, valvular pigmentation, network pharmacology, molecular docking, sirtuins, superoxide dismutase

## Abstract

**Objective:**

Angelica keiskei is a medicinal and edible plant that has been reported to possess potent antioxidant properties in several in vitro models, but its effectiveness on naturally aging organisms is still lacking. This study explores the antioxidant and health-promoting effects of Angelica keiskei in naturally aging mice.

**Methods:**

We treated 48-week-old mice with Angelica keiskei water extract (AKWE) 30 days, and measured indicators related to aging and antioxidants. In addition, we conducted network pharmacology analysis, component-target molecular docking, real-time PCR, and MTS assays to investigate relevant factors.

**Results:**

The results indicated that administration of AKWE to mice led to decrease blood glucose levels, improve muscle fiber structure, muscle strength, gait stability, and increase levels of glutathione and superoxide dismutase in serum. Additionally, it decreased pigmentation of the heart tissues. Angelica keiskei combats oxidative stress by regulating multiple redox signaling pathways, and its ingredients Coumarin and Flavonoids have the potential to bind to SIRT3 and SIRT5.

**Conclusions:**

Our findings indicated the potential of Angelica keiskei as a safe and effective dietary supplement to combat aging and revealed the broad prospects of medicinal and edible plants for addressing aging and age-related chronic diseases.

## 1. Introduction

Aging is a multidimensional biological process that entails alterations at various molecular and cellular levels. The theory of oxidative stress posits that in the early stages of aging, low levels of reactive oxygen species (ROS) can activate protective stress responses in the body [1,2]. However, as free radicals and ROS persistently accumulate, they induce DNA damage, such as DNA strand breaks and mutations at specific sites[3–6]. Meanwhile, oxidative stress can also result in phospholipid and protein (receptors and enzymes) peroxidation, ultimately leading to perturbations in cellular structure and consequent cell senescence/death [7–9]. Moreover, excessive oxidative stress is strongly correlated with aging-related diseases, including diabetes, atherosclerosis, and even cancer [10–13]. Gaining an understanding of how to maintain ROS at an appropriate physiological level within cells, holds significant research importance for the prevention of diseases and the delay of aging.

*Angelica keiskei* is a perennial herbaceous plant that belongs to the Umbelliferae family. It has been widely used as a medicinal herb or functional food due to its diverse pharmacological activities and safety. *Angelica keiskei* has shown potential for effects of anti-inflammatory, antihypertensive, antimicrobial, and anti-tumor properties [14,15]. Additionally, several studies have demonstrated that *Angelica keiskei* extracts possess anti-diabetic functional properties in mice models [16,17]. Ohnogi et al. have reported that *Angelica keiskei* extract treatment significantly reduced blood glucose levels in rats fed with high-fructose drink [18]. All of the previous data suggest a potential role of *Angelica keiskei* in improving aging-related symptoms. Coumarins, Chalcones and Flavonoids are the most abundant bioactive components in *Angelica keiskei* [14,19,20]. The most potent and abundant Chalcones in *Angelica keiskei* are 4-hydroxyderricin and xanthoangelol, which are well-known for their roles in tumor suppression, anti-diabetic, and anti-infective functions [21–24]. On the other hand, Flavonoids possess strong anti-inflammatory properties [25–27]. In addition, [28] they all have been recognized as potent antioxidants due to their superoxide scavenging activity [29–32]. Given their promising pharmacological properties, *Angelica keiskei* holds potential for being developed as a drug or health food that promotes healthy aging. Recently, a study on *Drosophila* has revealed a life span and health span extent effects of *Angelica keiskei* through insulin/IGF-1 pathway [33]. However, there is still insufficient *in vivo* evidence on mammals available to fully support this conclusion.

The natural aging model of mammals is frequently employed for evaluating the effectiveness of anti-aging medications. Previous studies have effectively validated the positive impacts of metformin, rapamycin, NMN, and other compounds on the lifespan and healthspan of mice in the natural aging model [34–39]. Building upon the preliminary research findings from *Angelica keiskei*, we conducted a first experiment to evaluate the influence of *Angelica keiskei* supplementation on the health of naturally aging mice and explored the underlying mechanisms.

## 2. Materials and methods

### 2.1. Preparation of *Angelica keiskei* water extract

The dried stem and leaves of *Angelica keiskei* were minced and mixed with twelve times the quantity of water. Reflux extraction was conducted two times with a duration of 1.5 h for each extraction. The extracted solution was filtrated and subsequently combined. The extract was concentrated under reduced pressure until it reached a relative density of 1.25–1.30 (at 60°C). The concentration process should occur at temperatures ranging from 40 to 90°C, while maintaining a vacuum degree of 0.04–0.1 MPa. The described procedure will result in the desired paste. The paste was collected using vacuum belt drying, with a heating plate temperature set between 105 and 120°C and a vacuum level of 0.04–0.1 MPa maintained. It is imperative to keep the moisture content of the dried paste below 5%. The dried paste was pulverized using an 80 mesh sieve, and the resulting powder was collected for further use. Approximately 1 g of fresh stem and leaves from *Angelica keiskei* can yield about 96.5 mg of dry powder. A quantity of 3.23 g of dry powder can produce 1 g of *Angelica keiskei* water extract powder.

### 2.2. Ultra-high performance liquid chromatogram analysis (UPLC)

The UPLC detection was performed using a Waters ultra-performance liquid chromatography system. The chromatographic column used was ZORBAX SB-C18, with dimensions of 2.1 × 100 mm and particle size of 1.8 μm. The flow rate was set at 0.4 mL/min, and the detection wavelength was 320 nm. The injection volume was 1 μL, and the column temperature was maintained at 30°C. The mobile phase consisted of solvent A (methanol) and solvent B (0.1% formic acid solution).

**Table:** 

Time(min)	A%	B%
0.00	5.0	95.0
10.00	25.0	75.0
25.00	25.0	75.0
27.00	30.0	70.0
38.00	80.0	20.0
48.00	80.0	20.0
48.50	90.0	10.0
53.50	90.0	10.0
54.00	5.0	95.0
60.00	5.0	95.0

### 2.3. Animal maintenance

Three-month-old and twelve-month-old male C57BL/6 mice were purchased from Beijing Weitong Lihua Experimental Animal Technology Co., Ltd. (Beijing, China). The animals were maintained in a specific-pathogen-free (SPF) condition room with a 12-hour light/dark cycle. They were housed at an ambient air temperature of 23 ± 2°C with a humidity level of 55%. The mice had ad libitum access to distilled water and rodent chow. Twelve-month-old mice with similar body weight were randomly grouped into middle-aged control and treatment groups based on their serum malondialdehyde (MDA) level. The three-month-old mice were considered as a young control. The treatment groups were given with AKWE daily by oral gavage. According to the National Health Commission of China, the recommended daily consumption of fresh *Angelica keiskei* for human is ≤50 g/day. The dry matter content of *Angelica keiskei* is 9.65% in our experiment. Therefore, the daily consumption of *Angelica keiskei* dry matter for human should be less than 4.825 g/day. We choose 4 g/day as the maximum amount, which proximally equals to 0.0667 g/kg/day per person, calculated based on a body weight of 60 kg. According to the principle of conversion based on surface area (mg/m^2^), the mouse equivalent dose (mg/kg body weight) is approximately 10 times that of the human dose. Therefore, we chose low, medium and high dosages of dried powder of AK for 0.333, 0.667, and 1.333 g/kg/day respectively, which equals to 103, 207, and 413 mg/kg/day for the AKWE (3.23 g of dry powder can produce 1 g of AKWE powder). AKWE powder is dissolved in distilled water. The middle-aged control group was administered an equal amount of water daily. After 30 days of AKWE administration, the C57BL/6J mice underwent biomolecular testing and pathological examination. The animal experiments were conducted in accordance with the ethical guidelines set by the Ethics Committee of Hebei Yiling Pharmaceutical Research Institute (No. TTN-2023013).

### 2.4. Blood glucose test

The serum samples were obtained by centrifuging the blood at 3500 rpm for 10 min. Blood glucose measurement was conducted using the Siemens advia2120i automatic hematology analyzer (Siemens Co., Ltd).

### 2.5. Grip strength testing

The grip strength tester (Columbus Instruments Co., Ltd) was calibrated and the force unit was set to kilogram-force (KGF) before the experiment starts. Mice were carefully positioned on the grip strength test mesh and were gently drawn backwards. The value of grip strength was recorded when the mouse's forelimbs released from the grip testing grid. The test was performed three times, and the highest value achieved is retained.

### 2.6. Gait analysis

Gait analysis was conducted using the DigiGait imaging and analysis system (Mouse Specifics, Inc., Framingham, MA, USA). During the experiment, mice were placed on a treadmill with a speed adjusted to 14 cm/second. A high-speed camera was employed to record the animal's movement. Gait parameters such as gait symmetry and swing duration coefficient (Swing Duration CV) were assessed using software provided with the instrument.

### 2.7. HE staining

Muscle and heart sections (4 μm) were stained with hematoxylin and eosin (H&E) to observe morphological changes. Briefly, the sections underwent deparaffinization with xylene, followed by rehydration using alcohol gradients and distilled water. Subsequently, the sections were stained with hematoxylin for 10 min. After differentiation and rinsing, the nucleus was counterstained with eosin for 1 min. Morphological changes were then observed using a Nano Zoomer-SQ digital slide scanner manufactured by Hamamatsu in Japan.

### 2.8. Lipid and protein peroxide detection

Serum samples were collected by centrifuging the blood at 3500 rpm for 10 min. The levels of protein carbonyl, glutathione (GSH), superoxide dismutase (SOD), and malondialdehyde (MDA) were measured using assay kits obtained from Nanjing Jiancheng Bioengineering Institute (Nanjing, China), Solarbio (Beijing, China), and Biyuntian (Shanghai, China).

### 2.9. MTS assay

HUVECs and C2C12 myoblasts were obtained from Procell Life Science & Technology Co., Ltd. (Wuhan, China). Cells reached about 80% confluence in the logarithmic growth phase were seeded into 96-well plates with a density of 6 × 10^4^ cells per milliliter respectively. For cytotoxicity test, *Angelica keiskei* extracts with different concentrations (dissolved in 0.1% DMSO) were added into HUVECs and incubated in 5% CO_2_ incubator (Thermo, USA, 311) at 37°C for 72 h. For antioxidation test, AKWE with different concentrations were added into H_2_O_2_ (800μmmol) pretreated C2C12 myoblasts. The 0.1% DMSO group was set as the solvent control group. The viabilities of HUVECs and C2C12 myoblasts were determined using CellTiter 96® AQueous One Solution Cell Proliferation Assay (MTS; Promega, Madison, WI, USA). Absorbance was detected using a microplate reader (infiniteM200pro, TECAN, Switzerland).

### 2.10. Quantitative real-time PCR

Total RNA was extracted using the TransZol Up Plus RNA Kit (TransGen Biotech, Beijing, China, ER501-01) and reverse transcribed with the GoScript™ Reverse Transcription System (Promega, Madison, USA, A5001). The cDNA products were then used as templates for qPCR with the MonAmp™ ChemoHS qPCR Mix kit (Monad, Suzhou, China, MQ00401S). The primer sequences are listed as follow.

**Table:** 

ID	Primer name	Sequence (5″ to 3″)
1	β-Actin-F	GTGCTATGTTGCTCTAGACTTCG
2	β-Actin-R	ATGCCACAGGATTCCATACC
3	SOD1-F	AACCAGTTGTGTTGTCAGGAC
4	SOD1-R	CCACCATGTTTCTTAGAGTGAGG
5	SOD2-F	CAGACCTGCCTTACGACTATGG
6	SOD2-R	CTCGGTGGCGTTGAGATTGTT

### 2.11. Network pharmacology analysis

Target genes of the herb *Angelica keiskei* were extracted using the Traditional Chinese Medicine Systems Pharmacology (TCMSP) database and analysis platform (https://temsp-e.com/temsp.php). Criteria were established to ensure the quality of the target genes, requiring an oral bioavailability (OB) of ≥30% and drug likeness (DL) of ≥0.1. The ETCM database (http://www.tcmip.cn/ETCM/index.php/Home/Inde/) and CTD database (https://ctdbase.org/) were utilized to supplement and eliminate redundancy in the obtained target genes. In addition, the GeneCards database (http://www.genecards.org) was used to search for disease genes related to aging using the keyword ‘Aging’, while the CTD database was used with the keyword ‘Antioxidant’ to search for genes related to oxidative stress. Subsequently, the overlapping genes between the target genes of the *Angelica keiskei* components and those related to aging and oxidative stress were selected using Cytoscape 3.9.0 software. Further analysis was conducted using the ClusterProfile R package for GO enrichment analysis and KEGG pathway enrichment analysis of the overlapping genes, resulting in the generation of enrichment bar plots and bubble charts.

To explore protein–protein interactions, a visual network graph of the drug component-target pathways was constructed using Cytoscape 3.9.0. The overlapping genes were then submitted to the STRING 12.0 database (http://string-db.org) with Homo sapiens selected as the species, and a confidence threshold above 0.7 was set. Through the exclusion of unconnected target genes, a protein–protein interaction (PPI) network of the overlapping genes was obtained. The data was imported into Cytoscape 3.9.0 to generate a protein–protein interaction (PPI) network graph and calculate the network's topological parameters.

### 2.12. Component–target molecular docking

Molecular docking method was used to virtual evaluate the binding affinity between the compound with target proteins, which might provide a reference for further experimental verification. AutoDock Vina 1.1.2 is an open-source program for molecular docking and virtual screening. Compared with AutoDock 4, its average accuracy of binding mode prediction is much higher [28]. Compounds of SDF files downloaded from the PubChem database (https://pubchem.ncbi.nlm.nih.gov/) [40]and through the Open Babel against 2.4.1[41] into MOL2 format. The crystal structure of Catalase (PDB ID: 8HID), Sirtuin 3 (PDB ID: 4BN4) and Sirtuin 5 (PDB ID: 7X3P) was downloaded from PDB database (https://www.rcsb.org/)[42]. Small molecules were energy minimized by using PyRx-0.8 software. Ligands and receptors were prepared according to the AutoDock Vina 1.1.2 tutorial. For each structure, we removed water molecules, added nonpolar hydrogen, calculated Gasteiger charges, and saved them in the PDBQT format. Generally, the lower the Vina score, the higher the Affinity between ligand and receptor. The general selection threshold is binding energy ≤ −5 kcal/mol [43,44]. Finally, Pymol software was used to visualize the interaction mode between compound and target proteins.

### 2.13. Statistics

All statistical analyzes were performed using IBM SPSS 22. Data were checked for normal distribution (Shapiro–Wilk test) and homogeneity of variance (Levene’s test for equality of variance) before choosing the appropriate statistical test. One-way analysis of variance (ANOVA) was used to evaluate the significant difference in normally distributed data. The Kruskal–Wallis test was used for nonnormally distributed data analysis. *P* values below 0.05 were considered significant for all analyzes. All data are presented as the mean ± standard deviation (SD).

## 3. Results

**Figure 1.**
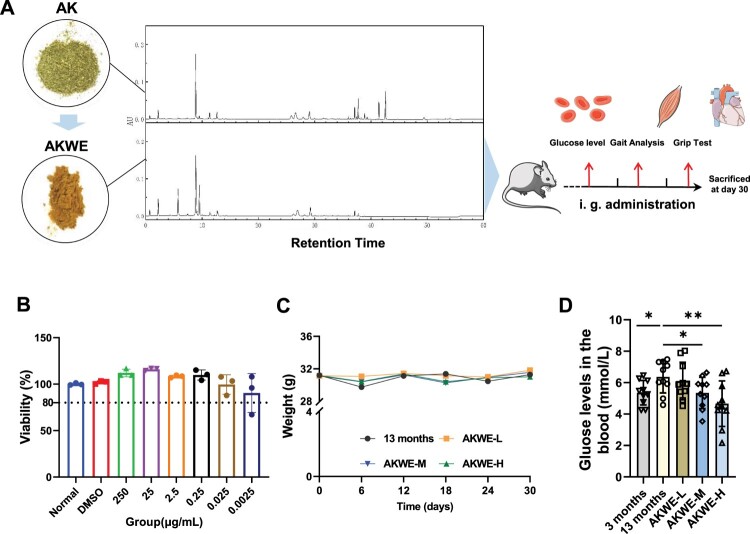
Effects of AKWE on body weight and blood glucose level of middle-aged mice. (A) The TUPLC-Q Orbitrap HRMS negative ion mode total ion flow chromatography of AKWE. (B) The viability of AKWE treated HUVECs. *n* = 3. (C, D) The weight growth curve and the blood glucose levels of AKWE treated mice. *n* = 10. *p < 0.05. Error bars indicate SD.

Initially, we prepared a water extract of *Angelica keiskei* using two extraction methods. UPLC analysis indicated that most of the liquid-phase peaks in AKWE and the liquid-phase peaks of the original herbal powder exhibit consistent retention times and similar peak heights, suggesting a similarity in components between the two materials (Figure 1A). MTS assays demonstrated that concentrations of AKWE ranging from 0.0025 to 250 µg/mL did not significantly affect the viability of HUVEC cells (Figure 1B). Subsequently, a 30 d administration experiment was conducted on 12-month-old mice to examine the effects of *Angelica keiskei* on the natural aging process. The AKWE at all dosages did not result in weight changes compared to the solvent control group throughout the 30 days administration period (Figure 1C). This result together with the MTS data collectively confirmed the safety of AKWE. Interestingly, high dose of AKWE significantly reduced random blood glucose levels in aged mice (mean blood glucose of aged mice: 6 mmol/L, mean blood glucose of the treatment group: 4.5 mmol/L, Figure 1D). Previous studies have documented the hypoglycemic effects of *Angelica keiskei* in rats with diabetes or obesity [18]. However, this study presents novel evidence of the hypoglycemic effects of AKWE in naturally aging animals.

**Figure 2.**
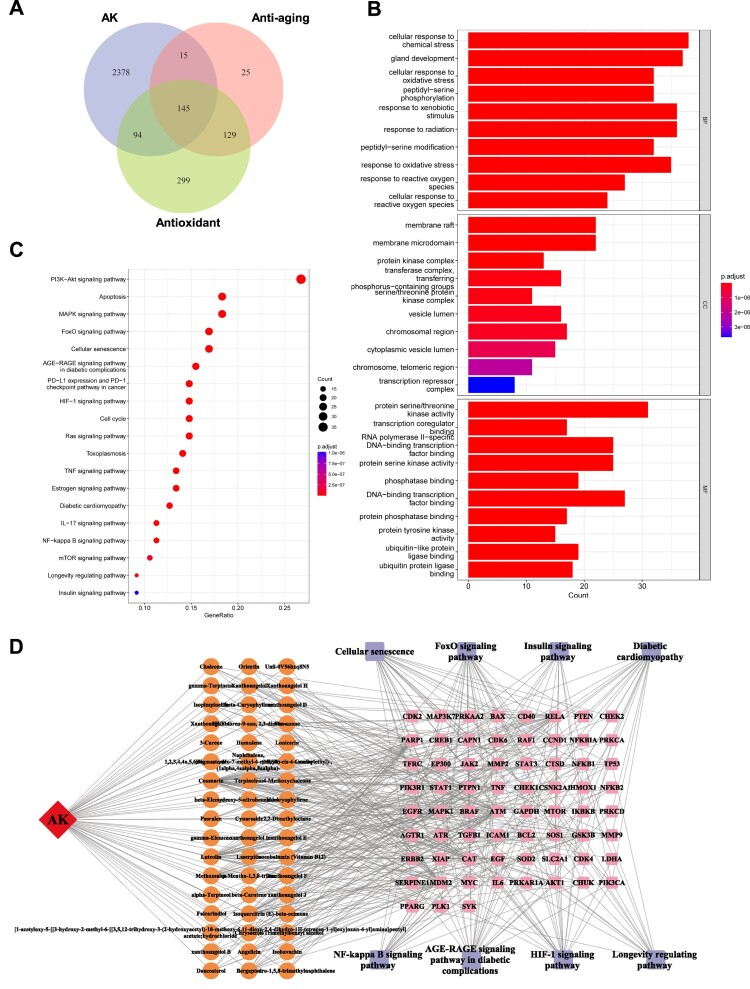
Network pharmacological analysis of AKWE on the antioxidative effects. (A) Venn diagram depicting the intersection of AK target genes, anti-aging genes, and antioxidant genes. (B) GO analysis of the intersectional gene set. (C) KEGG analysis of the intersectional gene set. (D) The interconnectedness between active ingredients, target genes, and associated pathways.

*Angelica keiskei* contains compounds that can be mainly categorized into three groups: Flavonoids, Chalcones, and Coumarins (Table 1) [14,19,20]. In order to investigate its medicinal efficacy during aging process, we extracted 2632 target genes for *Angelica keiskei* components from the TCMSP database (https://tcmsp-e.com/tcmsp.php). In addition, we obtained 314 genes associated with anti-aging potency and 667 genes with antioxidant properties from the Genecards database. Comparison of these collections revealed that 5.5% of the targets affected by *Angelica keiskei* compounds were involved in alleviating cellular oxidative stress and mitigating the aging process (Figure 2A). The GO functional enrichment analysis of the intersecting genes also indicated a significant enrichment of target genes in 365 biological processes (genes with more than 20 involvement), particularly those that respond to external stimuli (Figure 2B). Furthermore, these genes showed enrichment in 59 molecular functions (genes with more than 15 involvement), with a majority being specifically enriched in protein kinase activities and DNA transcription. The KEGG pathway enrichment analysis of the intersecting genes indicated that these potential target genes were involved in various essential anti-aging pathways, such as FoxO signaling, cellular senescence, the HIF-1 pathway, and the TNF-α pathway (Figure 2C). Additionally, some target genes were found to be involved in the pathogenic regulation of diabetes, which is consistent with previous reports indicating the therapeutic effects of *Angelica keiskei* on diabetes mellitus (Figure 2C). It is worth noting that certain target genes may also play a role in the AGE-RAGE pathway (Figure 2C). When advanced glycation end products (AGEs) bind to their receptor, known as the receptor for AGEs (RAGE), it leads to the generation of NAPDH and an increase in oxidative stress. This then activates the NF-κB signaling pathway, ultimately stimulating the production of cytokines and growth factors, resulting in cellular and tissue damage [45].

**Table 1.** Ingredients of *Angelica keiskei*.

Name	CID	ChEMBL_ID
Pteryxin	5281425	CHEMBL1884860
4'-methoxychalcone	641819	CHEMBL105496
chalcone	637760	CHEMBL7976
arabinogalactan	24847856	#N/A
Cerotin	14542802	#N/A
Daucosterol	5742590	CHEMBL506678
Stigmasterol	5280794	CHEMBL400247
isoquercetin	5280804	CHEMBL250450
Falcarindiol	5281148	CHEMBL69018
selinidin	66808	CHEMBL3660344
Isobavachalcone	281255	CHEMBL512908
Cynaroside	5280637	CHEMBL233929
Lonicerin	5282152	CHEMBL452979
Vitamin B12	5460135	#N/A
Xanthoangelol	643007	CHEMBL494083
4-hydroxyderricin	438503	CHEMBL111208
Xanthoangelol B	10409180	CHEMBL494082
Xanthoangelol C	15731056	#N/A
Xanthoangelol D	11302670	CHEMBL1711961
Xanthoangelol E	10022050	CHEMBL1718454
Xanthoangelol F	6479088	CHEMBL1722838
Isobavachin	193679	CHEMBL491534
Xanthoangelol H	6479089	CHEMBL1733408
Xanthoangelol I	11524001	#N/A
Xanthoangelol J	11675925	CHEMBL491512
Isopimpinellin	68079	CHEMBL140796
Methoxsalen	4114	CHEMBL416
Chrysoeriol	5280666	CHEMBL214321

Reported compounds in *Angelica keiskei* includes Coumarin compounds and Flavonoid compounds.

**Figure 3.**
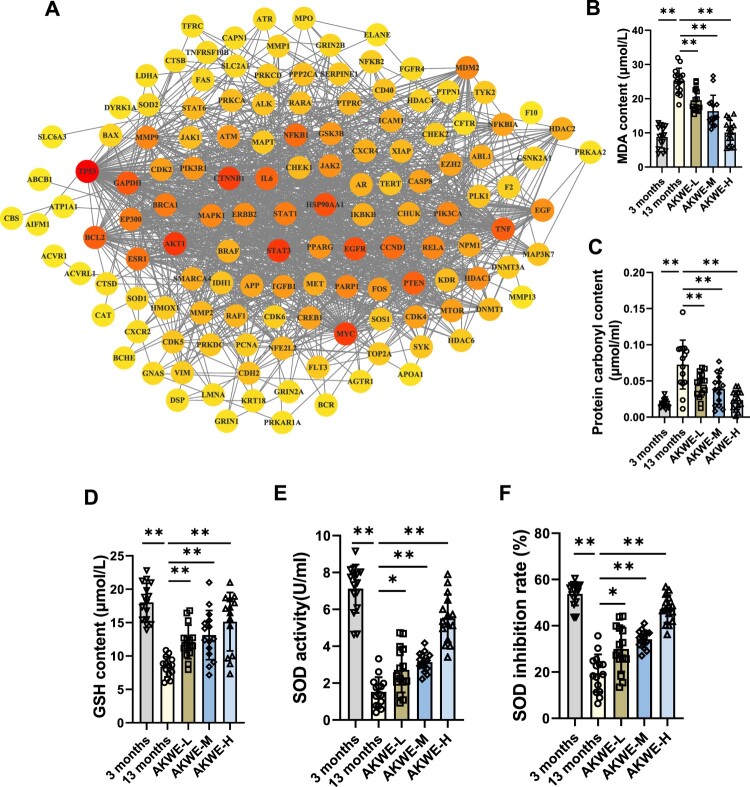
Detection of antioxidants in AKWE treated mice. (A) The PPI network of intersectional gene set. (B, C) The contents of serum MDA and protein carbonyl of AKWE treated mice. (D) The serum GSH content of AKWE treated mice. (E, F) The activity and inhibition rate of serum SOD in AKWE treated mice. *n* = 15. *p < 0.05, **p < 0.01. Error bars indicate SD.

Figure 2D depicts the interconnectedness between active ingredients, target genes, and associated pathways. This network comprises indicative genes associated with antioxidant responses (SOD and CAT), as well as the master regulators in the inflammation pathway (NF-κB and TNF-α). In order to gain a better understanding of the regulatory relationships between these target genes, we performed protein–protein interaction (PPI) network analysis (Figure 3A). Interestingly, the tumor-associated transcription factor MYC and TP53, were shown to be affected by *Angelica keiskei* and play central roles in this network, providing a potential new insight into the mechanisms of anti-aging by *Angelica keiskei*. To further confirm the *in vivo* antioxidant activity of *Angelica keiskei*, we evaluated the levels of lipid peroxidation and protein carbonylation in the aged mouse blood. The results revealed that *Angelica keiskei* extracts at all dosages significantly decreased the levels of MDA and protein carbonyl products in the serum samples of aged mice, compared to the solvent control group (Figure 3B,C). Moreover, this effect was observed to be dose dependent. Subsequently, we also measured the levels of glutathione content and SOD activity. The activity of superoxide dismutase (SOD) can be measured conveniently by using the highly water-soluble tetrazolium salt WST-1, known as 2-(4-iodophenyl)-3-(4-nitrophenyl)-5-(2,4-disulfophenyl)-2H-tetrazolium monosodium salt. When superoxide anion is reduced, WST-1 produces a water-soluble azo dye. The reduction rate is directly proportional to the activity of xanthine oxidase (XO) and is inhibited by SOD. In line with the reduced peroxide levels, the *Angelica keiskei* extract augmented the glutathione level in the blood, while also enhancing SOD activity and inhibition rate (Figure 3D–F).

**Figure 4.**
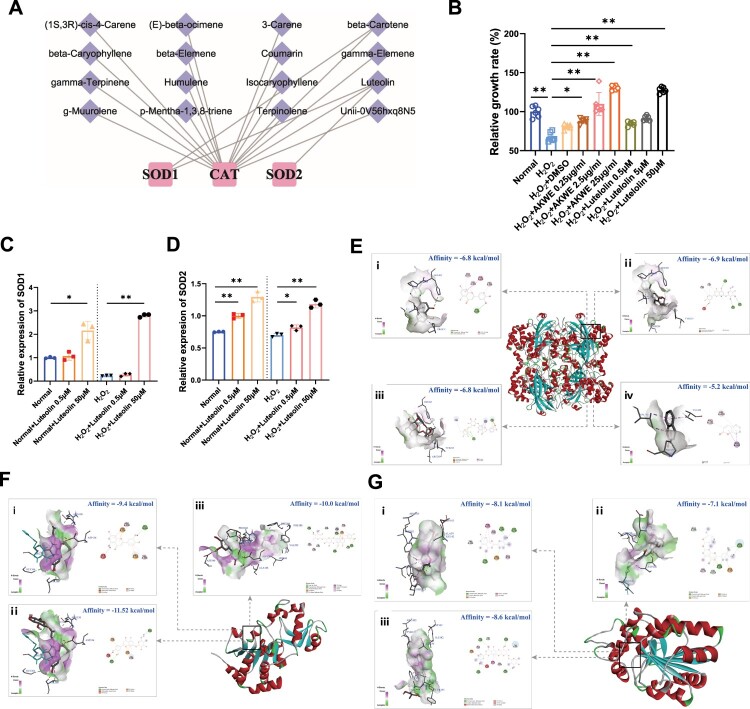
Bioactive ingredients of AKWE. (A) The ingredients of AKWE that potentially regulates antioxidative enzymes. (B) The MTS assay of AKWE and Luteolin treated H_2_O_2_ stressed C2C12 myoblasts. *n* = 3. (C, D) The mRNA levels of SOD1 and SOD2 in Luteolin treated C2C12 myoblasts. (E–G) The interaction mode between compounds and Catalase (E), SIRT3 (F), and SIRT5 (G); (i) Luteolin; (ii) Luteolin 7-O-glucoside; (iii) Luteolin 7-rutinoside; (iv) Coumarin. *p < 0.05, **p < 0.01. Error bars indicate SD.

To investigate the bioactive ingredients of AKWE, we examined the potential association between the compounds reported in *Angelica keiskei* and antioxidant enzymes. We noticed that the Coumarin and Luteolin have the potential to modulate several antioxidant enzymes, including SOD1, SOD2, and CAT (Figure 4A). To validate the antioxidant effect of Luteolin in the muscular aging process, we exposed C2C12 myoblasts to hydrogen peroxide. The MTS results demonstrated that oxidative stress caused a notable reduction in the viability of C2C12 myoblasts. However, incubation with varying concentrations of Luteolin effectively mitigated the decrease in cell viability and displayed a dose–dependent response (Figure 4B). The QPCR results revealed that Luteolin augmented the mRNA levels of SOD1 and SOD2 in mouse muscle cells following hydrogen peroxide treatment (Figure 4C,D). More interestingly, using molecular docking, we discovered that Coumarin, Luteolin, Luteolin 7-O-glucoside, and Luteolin 7-rutinoside can bind to the active regulatory pockets of SIRT3, SIRT5 and CAT (Figure 4E–G). The stability of component–target binding conformation is directly related to the binding energy, with lower binding energy indicating a more stable conformation. Therefore, a binding energy of ≤ −5.0 kJ/mol is used as a screening standard [43,44]. In our models, the binding energy of the target protein CAT with the core components Coumarin, Luteolin, Luteolin 7-O-glucoside, and Luteolin 7-rutinoside, and the target proteins SIRT3 and SIRT5 with the core components Luteolin, Luteolin 7-O-glucoside, and Luteolin 7-rutinoside are all below −5 kJ/mol. SIRT3 and SIRT5 are acetyltransferases located in mitochondria, playing crucial roles in regulating SOD activity. The high affinity of SIRT3, and SIRT5 for the core components suggests that they may be key targets for the antioxidative effect of AKWE. Collectively, our findings suggested that the antioxidant activity of AKWE is at least partially derived from Flavonoids such as Luteolin and these substances may regulate the expression and activity of antioxidant enzymes through multiple pathways.

**Figure 5.**
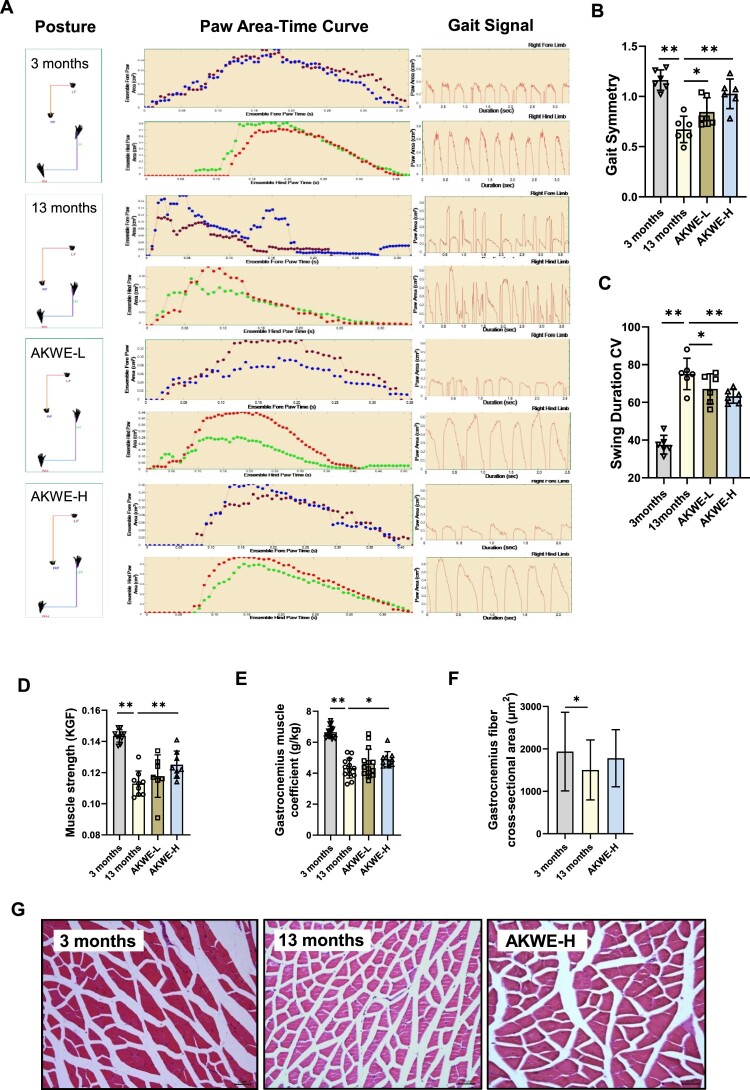
AKWE improves the locomotor ability of middle-aged mice. (A) The posture, paw area-time curve and gait signal of AKWE treated mice. (B) The gait symmetry and (C) swing duration CV of AKWE treated mice. *n* = 6. (D–F) The muscle strength, muscle coefficient, and muscle area of AKWE treated mice. (G) Representative images of H&E staining of gastrocnemius muscle. *p < 0.05, **p < 0.01. Error bars indicate SD.

One of the most notable characteristics of mammalian aging is the impaired functionality of the musculoskeletal system, including reduced strength, endurance, and coordination. Gait analysis, a well-established method for evaluating locomotor abilities in animals, demonstrated that the water extract of *Angelica keiskei* enhanced gait stability and reduced swing amplitude in middle-aged mice (Figure 5A–C). Additionally, significant improvements in grip strength were observed among middle-aged mice treated with high dose of AKWE compared to the control solvent group (Figure 5D). These findings suggest that AKWE effectively improves locomotor abilities in middle-aged mice, indicating a healthy aging process. Analysis of gastrocnemius muscles from middle-aged mice indicated that high dose of *Angelica keiskei* treatment caused a significant increase in muscle weight and an improving trend in muscle fiber structure (Figure 5E–G).

**Figure 6.**
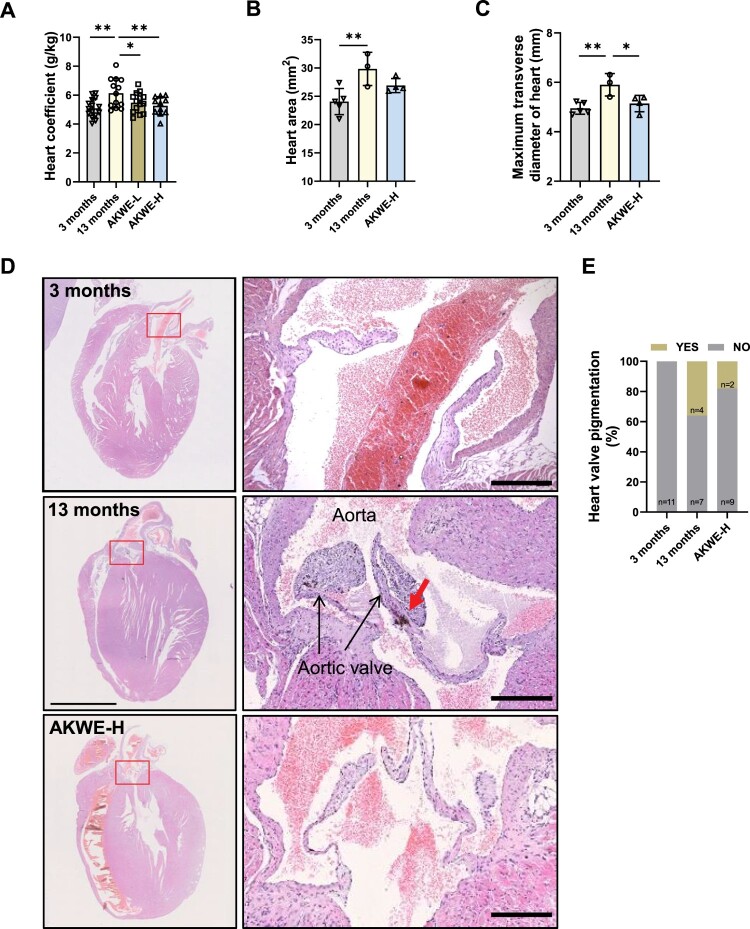
AKWE reduces the pigmentation of middle-aged mice heart. (A) Heart coefficient of AKWE treated mice. (B-E) Area, diameter, representative images of H&E staining of the heart, and ratio of valve pigmentation of AKWE treated mice heart. Red arrow indicates valve pigment deposition. *p < 0.05. Error bars indicate SD.

**Figure 7.**
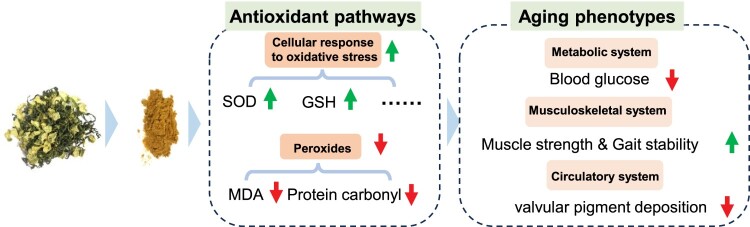
Summary figure of AKWE on the improvement of mice aging phenotype. AKWE exerts antiaging effects by targeting multiple redox signaling pathway, leading to an improvement in aging mouse phenotype.

Interestingly, instead of observing significant ameliorations in the physiological structure of aged skeletal muscle, a decrease in the heart coefficient of mice was observed following administration of AKWE (Figure 6A). Furthermore, the heart areas and diameters in the AKWE-treated groups also showed decreasing trends, suggesting a reduction in cardiac hypertrophy with *Angelica keiskei* treatment (Figure 6B,C). More notably, the high dosage of AKWE demonstrated a significant reduction in lipofuscin deposition in the aortic valves (Figure 6D,E). Lipofuscin is a polymer composed of peroxides and unsaturated fatty acids that primarily accumulates in non-neuronal cells, including cardiac myocytes and neurons. Abnormal lipofuscin deposition is linked to age-related oxidative stress, impaired lysosomal degradation, extracellular excretion, and elevated autophagy. Previous studies have identified lipofuscin deposition as one of the most common age-related changes observed in cardiac tissue [46,47]. Additionally, this accumulation can lead to increased stiffness of the heart valves and impact the blood supply to the heart [48,49]. These results further confirmed that *Angelica keiskei* has inhibitory effects on oxidative damage *in vivo*. Together, these results serve as evidence that the *Angelica keiskei* extract possesses the potential to mitigate oxidative stress in aging animals (Figure 7).

## 4. Discussion

This study is the first investigation of *Angelica keiskei* on improving health status of aging mammal. Upon comparing the potential targets of *Angelica keiskei* components with antioxidant proteins, we found evidence suggesting that *Angelica keiskei* may exert its antioxidant effects through SOD, CAT, HMOX1, and other redox signaling pathways (Figures 2 and 3). This hypothesis is strongly supported by the decrease in protein and lipid peroxide levels, as well as the increase in antioxidants, in the blood of mice treated with *Angelica keiskei* (Figure 3). Exposure to oxidative stress can lead to various modifications in proteins, including oxidation, cross-linking, and breakage. These modifications can hinder protein function, resulting in cellular dysfunction and tissue damage. The accumulation of damaged proteins is linked to the aging process and frequently seen in age-related diseases like Alzheimer's disease and Parkinson's disease. Conversely, lipids are the primary constituents of cell membranes and play a critical role in preserving cell integrity and function. Lipid peroxidation can occur due to oxidative stress, leading to the formation of lipid peroxides. Highly reactive, lipid peroxides can trigger a cascade of lipid damage. Excessive lipid peroxidation disrupts cell membrane structure and function, impacting cellular processes and promoting the development of various diseases, such as cardiovascular disease and neurodegenerative diseases. In the context of aging, alterations in protein and lipid peroxide levels, along with increased antioxidants, indicate a diminished capacity to accumulate oxidative stress and repair oxidative damage over time, resulting in an imbalance between oxidative stress and antioxidant defense. Our results suggested that *Angelica keiskei* is a potential anti-aging nature herbs with strong antioxidant effects. Although 13 to 15-month-old mice are generally considered as middle-aged mice, Davis et al reported that mice at the age of 15 month displayed impaired muscular function and morphology compared to the young ones [50] and we observed the similar phenotypes in the 13-month-old mice. We demonstrated that *Angelica keiskei* enhances muscle strength and motor stability in the middle-aged C57BL/6 mice in a dose-dependent manner (Figure 5). Further investigation of the hearts demonstrated that *Angelica keiskei* improved cardiac structure of aging male C57BL/6 mice and reduces heart index (Figure 6). Based on the observed improvement in cardiac valve lipofuscin deposition following treatment with the plant extract, it is possible that *Angelica keiskei* may enhance the circulatory system in aging mice through antioxidant signaling pathways. However, it is importance to conduct more extensive testing on cardiovascular system to confirm the beneficial effects of AKWE. These tests can include the following aspects: electrocardiogram (ECG), exercise stress test, echocardiography, and magnetic resonance imaging (MRI). The electrocardiogram provides information about cardiac electrical activity, the exercise stress test assesses the cardiovascular system's function under load, echocardiography examines cardiac structure and function, and magnetic resonance imaging provides detailed cardiovascular system images.

Interestingly, although there is a noteworthy increase in the muscle weight index among elderly mice treated with AKWE, the extent of improvement is relatively minor (Figure 5). Likewise, AKWE does not demonstrate a substantial enhancement in the cross-sectional area of the gastrocnemius muscle (Figure 5). In contrast, AKWE reduces the incidence of cardiac valve pigmentation by nearly fifty percent (Figure 6). This outcome implies that AKWE may considerably ameliorate the cardiovascular function of aged mice. A possible explanation for the varying impact of *Angelica keiskei* on the gastrocnemius muscle and myocardium in mice could be due to the continuous exposure of myocardial cells to a significant quantity of fresh blood. This exposure enables the active ingredients present in *Angelica keiskei* to exert more potent antioxidant effects on these cells. Furthermore, previous research conducted a 22-year follow-up analysis on 1378 physically healthy participants, with a mean age of 79.3 ± 7.3 years, and found a correlation between higher cardiovascular risk burden in older adults and a faster decline in physical functions, including agility, gait, and hand strength [51]. It is possible that the enhancement of the musculoskeletal system by *Angelica keiskei* may be partially attributed to improved cardiovascular health. However, conducting more extensive cardiovascular system testing is necessary to ascertain the beneficial effects of AKWE. This should include cardiac color ultrasound, blood flow velocity, and endothelial cell function tests. Moreover, in-depth investigation into the mechanisms of action of AKWE is essential.

The *Angelica keiskei* is acknowledged for its superoxide scavenging abilities. A preliminary examination of the literature has uncovered the existence of diverse compounds, such as Flavonoids, Coumarins, and Chalcones, within *Angelica keiskei* [14,19,20]. These compounds have been reported to exhibit antioxidant potential. Flavonoids, in particular, are bioactive substances found widely in plants, fruits, and vegetables, known for their anti-inflammatory, antioxidant, and antibacterial effects. Experimental animal models have demonstrated the ability of Flavonoids to extend lifespan [52]. Network pharmacology analysis suggests that specific Flavonoids of *Angelica keiskei*, such as Luteolin and Isoquercitrin, can target antioxidant stress-related proteins like SOD1, SOD2, and HMOX1, thereby enhancing the body's resistance to oxidative stress. Additionally, a group of Coumarins were discovered to target TNF-α and IL-6, potentially exerting an anti-inflammatory effect. We demonstrated that, applying Luteolin to H_2_O_2_ stressed C2C12 myoblasts could effectively rescue their viability and increase the SOD expression. Interesting, in addition to the transcriptional regulation of SOD gene, Luteolin and its glycosylated derivatives also showed good affinity with CAT, SIRT3 and SIRT5 by molecular docking. The CAT-Luteolin complex is stabilized by a hydrogen bond at the amino acid residue HIS-305. Luteolin 7-O-glucoside forms three hydrogen bonds with SER-201, GLN-455, and TYR-215 in CAT. CAT and Luteolin 7-rutinoside bind through two hydrogen bonds at TYR-215 and ARG-203. Luteolin interacts with SIRT3 through a hydrogen bond at GLY-364. SIRT3 and Luteolin 7-O-glucoside bind through two hydrogen bonds at GLU-323 and GLY-364. The SIRT3-Luteolin 7-rutinoside complex is stabilized by four hydrogen bonds at the amino acid residues VAL-292, ALA-146, ARG-158, and ASP-346. SIRT5 binds to Luteolin through three hydrogen bonds at TYR-102, ILE-142, and ASP-143. The SIRT5-Luteolin 7-O-glucoside complex is stabilized by two hydrogen bonds at ASP-228 and ASN-226. Luteolin 7-rutinoside forms four hydrogen bonds with TYR-102, GLU-225, ASP-143, and VAL-253 in SIRT5. SIRT3 is the major mitochondrial nicotinamide adenine dinucleotide (NAD+)-dependent deacetylase, which deacetylates two critical lysine residues (lysine 68 and lysine 122) on SOD2 and promotes its antioxidative activity. SIRT5, on the other hand, desuccinylates and activates SOD1 to eliminate ROS. These data suggest a potential role of Luteolin in SOD activity regulation. However, the exact affinity between Luteolin and SIRT proteins needs to be determined further through the application of biolayer interferometry or surface plasmon resonance assay. Additionally, the transcriptional regulatory mechanisms of antioxidant proteins, such as SOD, including their influence on transcription factors like NRF2, require further exploration. On the other hand, previous studies have found gender-specific effects of *Angelica keiskei* ethanol extract on extending the lifespan of Drosophila melanogaster. These effects may be due to its involvement in the insulin/IGF-1 pathway. Similar gender-specific effects on lifespan have been observed in mice and humans when dietary restriction is reduced and the impact on the insulin/IGF-1 signaling pathway is considered. However, it is important to note that the active components present in the aqueous and alcoholic extracts of *Angelica keiskei* may vary significantly due to their different polarities. Therefore, the next step of this study should focus on identifying the active components separately in both types of extracts. Furthermore, additional research is needed to determine if the benefits of AKWE in the aging process of mice also exhibit gender specificity and whether they are mediated through the insulin/IGF-1 pathway.

Taken together, our research provides initial evidence that *Angelica keiskei* improves the aging phenotype of mammals through its antioxidant properties. Future research will focus on the potential and pharmacological mechanisms of the extract in treating age-related diseases, such as musculoskeletal and circulatory system disorders.

## Data Availability

The data used to support the findings of this study are available from the corresponding authors upon request.
